# Noble gases confirm plume-related mantle degassing beneath Southern Africa

**DOI:** 10.1038/s41467-019-12944-6

**Published:** 2019-11-05

**Authors:** S. M. V. Gilfillan, D. Györe, S. Flude, G. Johnson, C. E. Bond, N. Hicks, R. Lister, D. G. Jones, Y. Kremer, R. S. Haszeldine, F. M. Stuart

**Affiliations:** 1 School of GeoSciences, University of Edinburgh, Grant Institute, James Hutton Road, Edinburgh, EH9 3FE UK; 2 Isotope Geosciences Unit, Scottish Universities Environmental Research Centre, East Kilbride, G75 0QF UK; 3 Department of Civil and Environmental Engineering, University of Strathclyde, James Weir Building, Glasgow, G1 1XJ UK; 4 School of Geosciences, University of Aberdeen, Meston Building, Kings College, Aberdeen, AB24 3UE UK; 5 Council for Geoscience, 139 Jabu Ndlovu St., Pietermaritzburg, KwaZulu-Natal, 3200 South Africa; 6 British Geological Survey, Environmental Science Centre, Nicker Hill, Keyworth, Nottingham, NG12 5GG UK; 7 Present Address: Department of Earth Sciences, University of Oxford, 3 South Parks Rd, Oxford, OX1 3AN UK

**Keywords:** Geodynamics, Geochemistry

## Abstract

Southern Africa is characterised by unusually elevated topography and abnormal heat flow. This can be explained by thermal perturbation of the mantle, but the origin of this is unclear. Geophysics has not detected a thermal anomaly in the upper mantle and there is no geochemical evidence of an asthenosphere mantle contribution to the Cenozoic volcanic record of the region. Here we show that natural CO_2_ seeps along the Ntlakwe-Bongwan fault within KwaZulu-Natal, South Africa, have C-He isotope systematics that support an origin from degassing mantle melts. Neon isotopes indicate that the melts originate from a deep mantle source that is similar to the mantle plume beneath Réunion, rather than the convecting upper mantle or sub-continental lithosphere. This confirms the existence of the Quathlamba mantle plume and importantly provides the first evidence in support of upwelling deep mantle beneath Southern Africa, helping to explain the regions elevation and abnormal heat flow.

## Abstract (web summary)

The elevation and high heat flow of Southern South Africa has controversially been attributed to a mantle plume. Here, the authors link degassed CO_2_ to a non-degassed mantle source rather than the convecting upper mantle, confirming plume-related mantle melting.

## Introduction

A striking feature of the African continent is the ~1 km elevation of the eastern and southern African plateaus. This has been termed the African Superswell^1^, and is also manifest by the shallow bathymetry of the southeastern Atlantic Ocean basin^2^. Seismic imaging beneath the African continent has revealed a large anomalous zone of low seismic velocity^3^, similar to that identified beneath the Pacific^4^. Termed Large Low Shear Wave Velocity Provinces (LLSVP) or superplumes, these are imaged to extend upwards from the core-mantle boundary^5^. Mantle flow induced by these velocity anomalies has been proposed to dynamically support elevated crustal regions^6^. The high topography of the eastern African plateau and unusual bathymetry of the southeastern Atlantic basin has been attributed to recent thermal modification of the upper mantle associated with the East African Rift System^7^. Recent geophysical^2^ and geochemical^8^ studies have indicated that the deeply rooted African superplume is the primary cause of this mantle anomaly, and is a major contribution to the Cenozoic rifting and volcanism of eastern Africa.

However, it is currently unclear if the anomalous topography of southern Africa is supported by a thermal perturbation in the lithospheric mantle^9^, the sub-lithospheric upper mantle^10^, the lower mantle^11^, or a combination of all three^2^. Previous seismic studies of the upper mantle structure beneath southern Africa have recorded only a small decrease in seismic velocities within the sub-lithosphere mantle, indicating that a thermal anomaly is unlikely^2^. Alternative hypotheses for the uplift of the region include; heating of the lithosphere by the tail of a Mesozoic plume that was stationary beneath the area for more than 25 million years^9^, or that it is the result of buoyancy from the African superplume present in the lower mantle^11^.

The Lesotho-KwaZulu-Natal region exhibits the highest relief in southern Africa^12^ forming the southernmost part of the African Superswell. The region experiences active seismicity^13^ and the sedimentary record of the Durban Basin and other Cretaceous basins surrounding southern Africa provide evidence for deposits sourced from distinct pulses of uplift and erosion in the Turonian, Oligocene, mid-Miocene and Pliocene^14^. Offshore, the anomalous bathymetry^15^ and seamounts^13^ of the Mozambique Basin, have been linked to active mantle upwelling associated with the hypothesised Quathlamba mantle plume^13^. Onshore, this could also explain the seismicity^16^, anomalous topography^12^, small-scale volcanic activity^17^, thermal springs^18^, elevated geothermal gradient^19^ and active CO_2_ seeps^13,20^ of the region.

However, the nature of the upwelling mantle and whether it originates in the deep or shallow mantle is not understood, nor is the relationship to the underlying African superplume. The isotopic composition of the noble gases (He, Ne and Ar) are an established geochemical method of distinguishing between deep undegassed^21,22^ and shallow convective mantle sources^23^. The presence of a noble gas signature of the deep mantle source associated with the ongoing CO_2_ degassing would provide a measure of whether mantle upwelling is related to the deep-sourced African superplume^24^ as opposed to a shallow convection-driven process in the depleted upper mantle^8^.

Here, we show that whilst the ^3^He/^4^He are lower than a typical primordial mantle source of >8 R_A_ (where R_A_ is the ^3^He/^4^He of atmospheric air of 1.399 × 10^−^^6^), the Ne isotopic composition of the degassing mantle CO_2_ requires a deep mantle source, similar to that tapped by intraplate volcanism at Réunion^25^ or Kerguelen islands^26^, rather than the convecting depleted upper mantle. This confirms the existence of the previously hypothesised Quathlamba mantle plume^13^ and illustrates that even modest plume induced lithospheric mantle melting, which is yet to result in significant extrusive volcanism, has incorporated a noble gas signature of the deep mantle source. Our findings provide the first geochemical verification of ongoing deep mantle upwelling in Southern Africa and corroborates existing geophysical evidence that small-scale mantle plumes are emanating from the top of the African LLSVP in the region^24^.

## Results

### Natural CO_2_ degassing in Lesotho-KwaZulu-Natal

**Fig. 1:**
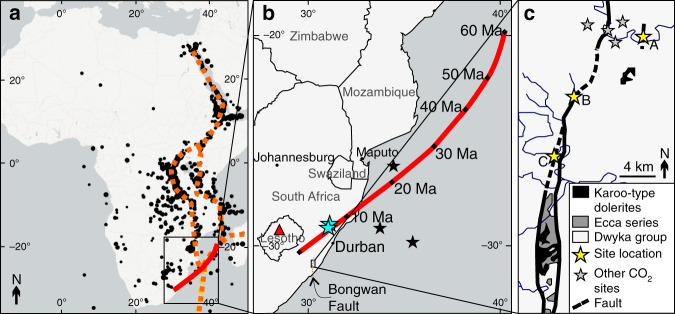
Location of study area and relevant geological features. **a** Map of the African continent illustrating depicting extent of panel 1b (inset box), the hypothesised track of the Quathlamba hotspot^13^ (red line), trace of the East African Rift System (EARS)^64^ (dashed orange lines) as picked out by earthquakes (black circles) between 2006 and 2016 (downloaded from the publicly available USGS database - https://earthquake.usgs.gov/earthquakes/search/ and plotted onto open source map tiles from the leaflet-extras R-library using Open Street Map tiles available via a CC-BY-SA licence from https://www.openstreetmap.org with country boundaries defined by Open Street Map). **b** Expanded map depicting study area (Panel c); the Bongwan fault trace (orange dashed line); the location of a small (1 m^3^) basaltic eruption that took place in Lesotho in 1983^17^ (red triangle); the locations of the Shu Shu and Lilani hot springs on the Tugela river (blue stars) which also exhibit minor CO_2_ degassing and hot water (up to 53 °C)^65^; the position of anomalous seafloor mounds in the northern Natal Valley^54^ (black stars) and the speculative track of the hotspot, based on African plate movement reconstructions, with corresponding proposed positions of the hotspot at the dates cited^13^. **c** Expanded map of Bongwan fault showing the location of the three sampling sites from this study marked as yellow stars (A – Baker Farm, B – Mjaja and C – Umtamvuna; location letters correspond to previous work^27^), the trace of the fault and the bedrock geology of the region. Historically reported^28,33^, but no longer accessible or existing seep locations are depicted as grey stars

CO_2_ gas seeps are common in areas of active or recent magmatism, and are frequently associated with fault-related fluid migration from depth^27^. Natural CO_2_ degassing is rare in South Africa; the natural cold CO_2_ seeps along the Ntlakwe-Bongwan fault in southern KwaZulu-Natal are the largest concentration of such phenomena. The fault was identified during geological mapping between 1911 and 1916^28^ with the seeps first described in 1923^29^ (Fig. 1a–c). The fault is expressed at the surface over ~80 km^30^ and is defined by a ~70 km wide arcuate zone of faulting that evolves southwards from an ENE-WSW to a north-south strike^31^. It is believed to be related to Gondwana rifting^32^ which commenced ~180 Ma and continues to the present (Fig. 1c).

The origin of the degassing CO_2_ is enigmatic, with initial work proposing a link to dissolution of carbonate rocks at depth by acidic groundwater^33^. Later δ^13^C and δ^18^O measurements of the CO_2_ indicated an origin from low temperature acidic groundwater reactions with carbonate rocks of similar composition to the Cambrian Matjies River Formation^20^ of the Western Cape Province. Carbonates of the nearby Marble Delta Formation were ruled out as a source, as their δ^13^C was found to be distinct from the δ^13^C_CO2_ measured in the exsolving CO_2_^20^, but this work did not take into account the potential fractionation of δ^13^C that would result from formation of a free-phase CO_2_ during dissolution of carbonate rock. Mantle melting associated with the hypothesised Quathlamba mantle hotspot has also been proposed as both a potential CO_2_ source, and a cause of a local thermal anomaly^13^.

Natural CO_2_ can have multiple origins, including; shallow biogenic processes, carbonate hydrolysis, deep burial related mechanical breakdown or thermo-metamorphism of carbonates and degassing of magmatic bodies^34,35^. Whilst δ^13^C_CO2_ can often resolve these sources, CO_2_-rich natural gases frequently exhibit values that overlap with the range of carbon from magmatic source and carbonate breakdown^34^, making it challenging to resolve their origin^36^. Noble gas isotopes are powerful tracers of the origin of CO_2_, particularly in identifying mantle contributions^37^. Primordial isotopes, such as ^3^He, originate in the Earth’s mantle and gases from the depleted upper mantle define a narrow range of CO_2_/^3^He (of 1 to 10 × 10^9^)^38–40^.

### Combining δ^13^C_CO2_ with CO_2_ and helium measurements to resolve CO_2_ origin

Here we combine new noble gas analyses of Bongwan CO_2_ and δ^13^C_CO2_ from six separate gas seeps, sampled from three locations along the Bongwan fault and associated splays (Supplementary Tables 1–2). δ^13^C_CO2_ range from −2.0 to −3.3‰ (V-PDB standard) in line with previous determinations^20^. ^3^He/^4^He ratios corrected for air (^3^He/^4^He_c_—see “Methods”) within the samples range from 3.6 to 4.5 R_A_. These are considerably above the atmospheric ratio (1 R_A_) indicating the presence of a significant amount of primordial ^3^He.

**Fig. 2:**
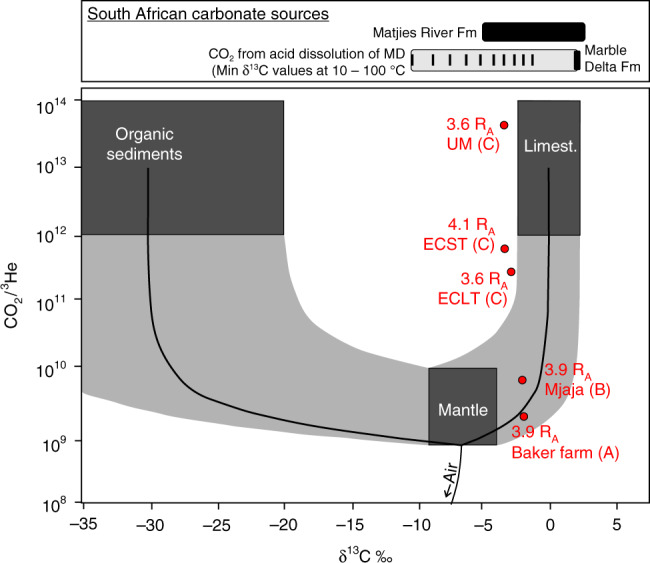
CO_2_/^3^He against δ^13^C for the Bongwan CO_2_ samples. Individual samples are plotted as red circles along with labels outlining their associated air-corrected ^3^He/^4^He_c_ in R_A_ (where R_A_ is the atmospheric ^3^He/^4^He of 1.399 × 10^−6^) and their location corresponding to Fig. 1 (A – Baker Farm, B – Mjaja and C – Umtamvuna). Mixing lines (black lines and grey shading) are shown for CO_2_ derived from the mantle, limestone and organic sediments^38^. End-member compositions cover the range of values^38,62^ (mantle CO_2_/^3^He = 1–10 × 10^9^; mantle δ^13^C = −9 to −4‰; Crustal CO_2_/^3^He = 1 × 10^12^–10^14^; Limestone δ^13^C = 0 ± 2‰; Organic sediment δ^13^C = −30 ± 10‰). The range of δ^13^C values for South African carbonate sources from the Marble Delta^20^, Matjies River Formation^20^, and the Transvaal Supergroup^66^ are provided. The extent of δ^13^C_CO2_ that would be produced by acid groundwater dissolution of Marble Delta Formation carbonate is also shown (grey box and black lines depicting the minimum δ^13^C_CO2_ that would result from dissolution of the formation carbonate between 10 °C (left) and 100 °C (right)) (see Methods). The trend between CO_2_/^3^He and δ^13^C_CO2_ are consistent with the mixing of mantle-derived CO_2_ with CO_2_ derived from the overlying Marble Delta Formation carbonates at up to 70 °C (see “Methods”). δ^13^C_CO2_ was not measured from the sample collected at the Umtamvuna River Spring and hence this sample cannot be depicted on the plot

^4^He exhibits the widest range in concentration compared to the other noble gases, from 1.48 × 10^−9^ (Umtamvuna Mound 2) to 9.62 × 10^−5^ cm^3^(STP)cm^−3^ (Baker Farm). ^20^Ne concentrations range from 4.01 × 10^−9^ to 4.13 × 10^−8^ cm^3^(STP)cm^−3^, with ^40^Ar ranging from 6.34 × 10^−6^ to 7.83 × 10^−5^ cm^3^(STP)cm^−3^. As with ^4^He concentrations, the lowest ^20^Ne and ^40^Ar values are exhibited by the CO_2_ sampled from Umtamvuna Mound 2, and the highest values are from the Baker Farm sample. CO_2_ from the Baker Farm and Mjaja seeps (A and B on Fig. 1c) exhibit CO_2_/^3^He of 1.88 and 6.78 × 10^9^, confirming a mantle origin (Fig. 2). The four seeps sampled at Umtamvuna exhibit considerably higher CO_2_/^3^He ratios. As ^3^He is inert and insoluble^37^, and there is no significant ^3^He in the crust^37^ (^3^He/^4^He_crust_ = 0.05 R_A_)^41^, the variation in CO_2_/^3^He is predominantly linked to the addition of ^3^He-poor CO_2_^42^.

Combining CO_2_/^3^He with δ^13^C_CO2_ allows organic sediment and limestone-derived CO_2_ to be distinguished from magmatic sources^38^ (Fig. 2). The trend between CO_2_/^3^He and δ^13^C_CO2_ are consistent with the mixing of mantle-derived CO_2_ with CO_2_ derived from the overlying Marble Delta Formation carbonates at up to 70 °C (see “Methods”). Based on the regional geothermal gradient of 30 °C/km^19^ and an average surface temperature of 14 °C^43^, we estimate that mixing occurred at depths of less than ~1900 m.

**Fig. 3:**
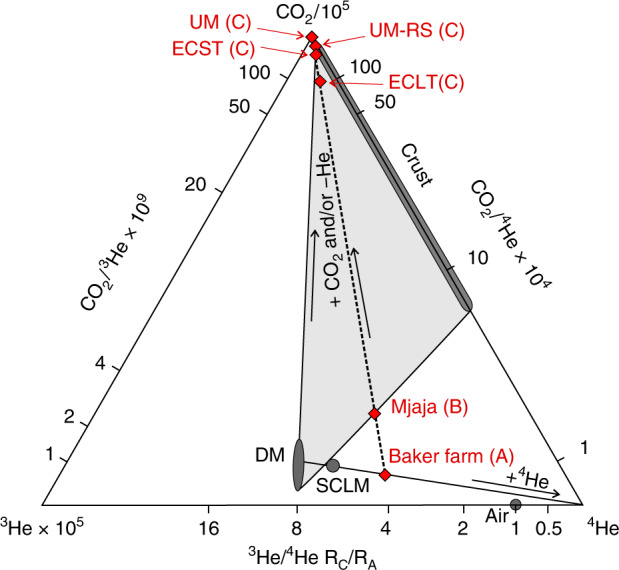
Ternary plot of CO_2_/^3^He, CO_2_/^4^He and ^3^He/^4^He of Bongwan CO_2_ samples. Relevant end-members, after^45^, with relative concentrations calculated for CO_2_, ^3^He, and ^4^He, based on these ratios. Scaling factors are introduced to the CO_2_ and ^3^He values, as labelled, to allow the full spread of data to be represented. The scaled relative concentrations are then normalised to a three-component system where CO_2_ + ^3^He + ^4^He = 100%, permitting direct comparison of the relative proportions of CO_2_, ^3^He, and ^4^He, regardless of absolute concentrations. Binary mixtures and loss or gain of any single component will plot as straight lines. The plot shows that the Bongwan CO_2_ samples have been influenced by two separate processes: (1) Mixing between depleted upper mantle (DM) (8 ± 1 R_A_)^46^ or sub-continental lithospheric mantle (SCLM) (6.1 ± 0.9 R_A_)^47^ and a radiogenic ^4^He component, and (2) Addition of a high-CO_2_, low-^3^He and ^4^He component and/or loss of both ^3^He and ^4^He relative to CO_2_. The Baker Farm sample lies on the first mixing line, and the addition of CO_2_ free of ^3^He and low in ^4^He and the effect of shallow preferential degassing of He relative to CO_2_ in the near-surface^48^ can account for the second trend. ECLT – East Cape Large Travertine, ECST – East Cape Small Travertine, UM-RS – Umtamvuna River Spring and UM – Umtamvuna

Linking CO_2_/^3^He, CO_2_/^4^He and ^3^He/^4^He in a ternary plot allows CO_2_ sources to be resolved^44,45^, permitting direct comparison of the relative proportions of CO_2_, ^3^He, and ^4^He, regardless of absolute concentrations (Fig. 3). Binary mixtures and loss or gain of a single component plot as straight lines on ternary plots. Figure 3 demonstrates that the Baker Farm gas requires the ingrowth/addition of 33 to 50 % radiogenic ^4^He, derived from the lithosphere, to mantle magmas, relative to the depleted upper asthenosphere mantle (DM) (8 ± 1 R_A_)^46^ or sub-continental lithospheric mantle (SCLM) (6.1 ± 0.9 R_A_)^47^, respectively. The remaining samples require the addition of ^3^He-poor CO_2_. The modest ^4^He required in this gas confirms a shallow crustal origin for this non-magmatic CO_2_ (also see Supplementary Fig. 1). The low ^4^He/^20^Ne in these samples contrasts with the higher values measured in the Mjaja and Baker Farm gases and implies that the CO_2_ has interacted with atmosphere-saturated groundwaters.

Recent work undertaken on CO_2_ seeps in Australia, similar to those at Bongwan, has highlighted that equilibration of mantle-sourced CO_2_ with atmosphere-saturated groundwaters, followed by solubility controlled fractionation during exsolution of the CO_2_ from the groundwater at CO_2_ seeps, can result in depleted He concentrations and elevated CO_2_/^3^He from values which are originally within the magmatic source range^48^. Given the wide range of ^4^He concentrations observed between the different seeps, it is likely that both mixing with crustal derived CO_2_ and solubility fractionation, resulting in a relative loss in He, have acted to produce the observed CO_2_/^3^He ratios at Bongwan (Fig. 3).

### Constraining the mantle source using Ne and Ar isotopes

The ^3^He/^4^He of the SCLM of the Karoo Large Igneous Province and the nearby volcanic ocean islands (Comores, Tristan da Cuna and Gough) are characterised by low ^3^He/^4^He (4.9–7.1 R_A_)^49^. Hence, the mantle source in the region may have ^3^He/^4^He that is lower than MORB, but is most likely to be above the highest measured ^3^He/^4^He_c_ of 4.27 R_A_. The CO_2_ degassed at the Bongwan seeps has migrated from the mantle through the crust and will have incorporated radiogenic He from the Precambrian metamorphic basement and sedimentary cover. Radiogenic ^4^He is required to account for CO_2_-He isotope systematics of the Baker Farm seep gas (Fig. 3) and can account for the ^4^He/^21^Ne* ratio of 1.23 × 10^7^ in the sample, below the crustal ratio of 1.71 × 10^7^ ^41^. Neon isotopes provide less ambiguous insights into the mantle source, enabling differentiation of depleted, convecting upper mantle (DM), the source of mid-ocean ridge basalts (MORB) and the primordial ^20^Ne-enriched mantle that is sampled by intraplate magmatism, the source of ocean island basalts (OIB)^50^.

**Fig. 4:**
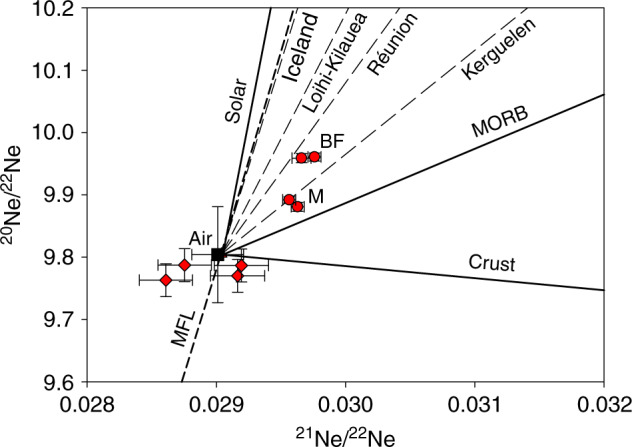
^20^Ne/^22^Ne plotted against ^21^Ne/^22^Ne for the Bongwan CO_2_ samples. Lower precision analysis performed on the MAP 215-50 mass spectrometer are plotted as red diamonds, higher precision analysis undertaken on the ARGUS VI mass spectrometer are depicted as red circles, with air plotted as a black square. 1σ errors associated with each measurement are provided along with mixing lines between air^67^, continental crust^41^, MORB^46^ and solar end members^62^ shown as solid black lines. The Kerguelen, Reunion, Loihi-Kilauea, and Iceland hotspots are depicted as thin dashed lines^46^ with the mass fractionation line (MFL) shown as a thick dashed line. The Baker Farm (BF) and Mjaja (M) gases plot above the established air-MORB mixing line^40,68^, plotting between the Kerguelen and the Reunion mantle source^25^, implying an undegassed mantle origin for the Bongwan CO_2_. The uncertainty differences reflect different analytical procedures and the ARGUS samples have been corrected for the contributions to ^21^Ne from NeH^+^, which is controlled by the amount of H_2_ present in the mass spectrometer during analysis^61^ (see “Methods”)

The Ne isotope composition of Baker Farm and Mjaja seeps show a clear mantle component, which is distinct from both atmospheric Ne and the mass fractionation line (Fig. 4). ^40^Ar/^36^Ar of the Mjaja and Baker Farm gases (550 ± 2 and 961 ± 4 respectively) are higher than the air value, consistent with a partial mantle origin of the non-atmospheric ^40^Ar. This is supported by the ^4^He/^40^Ar* (1.55 and 1.77 respectively) which are close to the mantle value of 2^37^. The remaining samples have atmospheric dominated Ne and Ar isotope compositions consistent with derivation of Ne and Ar from air-equilibrated groundwater. This corresponds to the crustal CO_2_ addition from groundwater and/or He loss due to degassing of CO_2_ from groundwater^48^, required to account for the elevated CO_2_/^3^He in these samples, compared to the more mantle-rich values of Mjaja and Baker Farm.

Importantly, the results of the high precision Ne analysis of Mjaja and Baker Farm seep gases do not plot on the MORB-air mixing line in Ne isotope space (Fig. 4). Instead they provide a clear indication that the mantle source of the CO_2_ is more primordial than that of the convecting upper mantle (Fig. 4). The duplicate high precision determinations of the Baker Farm and Mjaja CO_2_ samples overlaps with the trend defined by the Kerguelen^26^ and Réunion hotspots^25^, implying the ultimate source of the upwelling mantle is deep. The low ^3^He/^4^He of the Bongwan gases relative to Kerguelen (12.3 ± 0.3 R_A_) and Réunion (11.5–13.1 R_A_) would require the incorporation of crustal radiogenic ^4^He.

However, the ^3^He/^4^He of the sub-lithospheric mantle source of the nearby Karoo Large Igneous Province is cited as 7.03 ± 0.23 R_A_^51^ and the most proximal volcanic ocean islands (Comores, Tristan da Cuna and Gough) are characterised by low ^3^He/^4^He (4.9 to 7.1 R_A_)^51^. Hence, the mantle source in the region may have a ^3^He/^4^He that is not elevated above the MORB range (8 ± 1 R_A_), though is most likely to be above the highest measured ^3^He/^4^He_c_ of 4.5 R_A_. It is also possible that the He and Ne systematics of the mantle under southern South Africa are decoupled, as has been observed in the Icelandic and the Colorado Plateau mantle sources^23,52,53^. This decoupling was attributed to either more compatible behaviour of He during low-degree partial melting or more extensive diffusive loss of He relative to the heavier noble gases. Incorporation of crustal-radiogenic ^21^Ne to the Bongwan gases is also probable, but without constraint of the original Bongwan mantle ^3^He/^4^He this is impossible to determine.

## Discussion

The Bongwan CO_2_ seeps are located at the end of the hypothesised Quathlamba hotspot track. Hotspot migration has been proposed to explain chain of volcanic seamounts that track across the Mozambique Basin^13^, orientated in a direction that closely resembles reconstructions of the African plate movement (Fig. 1). Recently, anomalous 30 km elongate seamounts, have been identified within the Northern Natal Valley offshore of Durban^54^. The geospatial positioning of these could extend the East African Rift System southwards, but they are also within range and age of the proposed Quathlamba hotspot track (Fig. 1b). Furthermore, recent work has found that the seafloor adjacent to the Mozambican continental margin, and that of the central Mozambique Channel is 300 m and 1300 m shallower, respectively, than the conjugate basins in Antarctica, or than oceanic thermal subsidence models predict^15^. This has been attributed to the presence of thickened oceanic crust, linked to the passage of a mantle plume beneath the basin during the Paleogene^15^.

Plate movement reconstructions indicate that this hotspot moved under the continent approximately 10 million years ago (Fig. 1), coinciding with several periods of regional uplift^55^. The Ne isotope systematics of the CO_2-_rich gas seeps provide the first geochemical evidence that small volumes of melting is occurring beneath the continental lithosphere in the region. This confirms the existence of the previously hypothesised Quathlamba mantle plume^13^ and illustrates that even modest plume induced lithospheric mantle melting, which is yet to result in significant extrusive volcanism, has incorporated a noble gas signature of the deep mantle source. These findings provide the first geochemical verification of ongoing mantle upwelling in Southern Africa, confirming geophysical evidence that small-scale mantle plumes are emanating from the top of the African superplume^24^. Buoyant underplating of Southern Africa by the African superplume provides an explanation for the anomalous elevation, high heat flow, and how the Quathlamba mantle plume has incorporated deep-sourced mantle volatiles.

## Methods

### Fieldwork

The CO_2_ seeps were identified in the field as bubble streams in pools of water, rivers and wellbores (Fig. 1, Supplementary Table 1). Gas samples were collected in September 2015 by placing a plastic funnel over the site of the CO_2_ seep and flowing the gas through a 70 cm length of refrigeration grade copper tubing fitted with an exhaust hose to prevent turbulent back-mixing of air into the sample. The tubing was purged with the seeping gas for between 10 and 15 min before being clamped by a purpose built tube clamp at both ends to seal the copper tube with a cold-weld that is impervious to helium^36^. Tedlar sample bags were filled at each seep for stable isotope analyses. The Baker Farm borehole was sampled by sealing the well and using a soil gas probe to collect gas from as deep as possible within the well. A Geotechnical Instruments GA2000 portable gas analyser was then used to extract gas from the Baker Farm well, with the pump being connected downstream of the sampling apparatus. Further details on individual field sites and other surveys undertaken in the area are outlined in Supplementary Table 1 and in previous work^27,56^.

### Laboratory analysis

Bulk gas, stable isotope and noble gas analysis was undertaken at the Scottish Universities Environmental Research Centre (SUERC), using previously described techniques^57^. Bulk gas content as a percentage was determined using a Pfeiffer Vacuum QMS 200 quadrupole mass spectrometer with all seeps sampled exhibiting concentrations of >99% CO_2_. δ^13^C_CO2_ were measured using a VG SIRA II dual inlet isotope ratio mass spectrometer following established procedures^58^. Precision and reproducibility are typically better than ±0.2‰ for δ^13^C (Supplementary Table 2).

Noble gas analyses from all samples were performed on volumes of ~10 cm^3^ gas stored in copper tubes. Each sample was expanded to a titanium sublimation pump (900 °C) and a series of SAES GP50 ZrAl getters (250 °C) operating under ultra-high vacuum, following established procedures^57–60^. The isotopic composition of He, Ne and Ar of all six samples was measured using a MAP 215-50 mass spectrometer using established techniques^57–60^ (Supplementary Tables 2 and 3). Analytical errors are governed by the reproducibility of air calibrations, and for Ne, standard reproducibility was assessed using the best Gaussian fit to the probability density distribution of ^21^Ne/^22^Ne and ^20^Ne/^22^Ne ratios from 14 air calibrations, which is an objective way of filtering outliers. These samples were not corrected for any ^20^NeH^+^ contribution to ^21^Ne.

High precision analysis of Ne isotopes within the Baker Farm and Mjaja samples was undertaken in multi-collection mode using a ThermoFisher ARGUS VI using the following procedures. Each copper tube sample was mounted on the ultra-high vacuum line attached to the MAP 215-50 mass spectrometer, and subjected to the same clean up procedure as outlined above, following which they were trapped in a 2 L stainless steel cylinder. Approximately 100 cm^3^ of total gas was extracted from this cylinder to the ultra-high vacuum system attached to the ARGUS VI mass spectrometer as described in previous work^61^. The gas was exposed to another SAES GP50 ZrAl getter (held at 250 °C) for 15 min and then a liquid nitrogen-cooled charcoal finger (held at −196 °C) for 15 min to trap any remaining active gases along with Ar, Kr and Xe. Ne was then adsorbed on charcoal using an IceOxford cryopump (−243 °C, 20 min) while He was pumped away. Pure Ne was released at −173 °C and administered into the ARGUS VI low resolution mass spectrometer. The ARGUS clean-up and analysis procedure was undertaken twice for both the Baker Farm and Mjaja samples, and the results of the individual repeat measurements are plotted on both Fig. 4 and Supplementary Fig. 3, and listed in Supplementary Table 4.

Analysis of Ne isotopes followed procedures described in previous work^61^. Ne isotopes were multi-collected (^22^Ne^+^– H_2_, ^21^Ne^+^ – Axial, ^20^Ne^+^ – L2) on 10^12^ Ω Faraday amplifiers. Isobaric interferences of ^40^Ar^2+^ and ^44^CO_2_^2+^ were quantified using pre-determined singly/doubly charged ratios under measurement conditions and the in situ measurement of ^40^Ar^2+^ and ^44^CO_2_^2+^ during analysis on the CDD detector. The contributions of ^40^Ar^2+^ to the corresponding ^20^Ne peak were found to be ~0.6% and 0.07% for Baker Farm and Mjaja samples, respectively. Contributions of ^44^CO_2_^2+^ to ^22^Ne for Baker Farm and Mjaja were found to be 0.2% and 0.04%, respectively. The contribution of ^40^Ar^+^ and ^44^CO_2_^+^ to the overall uncertainty of Ne isotopic ratios was found to be below 0.01%. Other interferences (H_2_^18^O^+^, H^19^F^+^, ^65^Cu^3+^) were found to be negligible. The ^20^NeH^+^ contribution at m/z = 21 was determined using a pre-recorded calibration curve of ^22^Ne – ^22^NeH at a constant hydrogen level, that exceeded the level of ^22^Ne, where ^20^Ne of each sample was measured^61^. The measurement of ^22^NeH occurred at *m/z* = 23, corrected for ^46^CO_2_^2+^ and blank. ^20^NeH^+^ correction at *m/z* = 21 was found to be 0.96% (Baker Farm) and 1.94% (Mjaja). The contribution of NeH correction toward the overall uncertainty is ± 0.02%. Mass fractionation was corrected by the repeated analysis of air prior to and after analysis (*n* = 9) with the reproducibility of ^20^Ne/^22^Ne = 0.05% and ^21^Ne/^22^Ne = 0.11%.

### Data analysis

^3^He/^4^He were corrected for minor atmospheric air contributions using the measured ^4^He/^20^Ne, following the established formula:^37^

1 $$\left( {{\,}^3\mathrm{He}/{\,}^4\mathrm{He}} \right)_c = \frac{{\left( {{\,}^3\mathrm{He}/{\,}^4\mathrm{He}} \right)_s \times \left( {{\,}^4\mathrm{He}/{\,}^{20}\mathrm{Ne}} \right)_s/\left( {{\,}^4\mathrm{He}/{\,}^{20}\mathrm{Ne}} \right)_{\mathrm{air}} - \left( {{\,}^3\mathrm{He}/{\,}^4\mathrm{He}} \right)_{\mathrm{air}}}}{{\left( {{\,}^3\mathrm{He}/{\,}^{20}\mathrm{Ne}} \right)_s/\left( {{\,}^3\mathrm{He}/{\,}^{20}\mathrm{Ne}} \right)_{\mathrm{air}} - 1}}$$

Mixing curves shown in Fig. 2 are calculated after^62^ using E

2 $$\left( {\frac{{{\,}^{13}{\mathrm{C}}}}{{{\,}^{12}{\mathrm{C}}}}} \right)_{\mathrm{Obs}} = {\mathrm{A}} \ast \left( {\frac{{{\,}^{13}{\mathrm{C}}}}{{{\,}^{12}{\mathrm{C}}}}} \right)_{\mathrm{A}} + {\mathrm{B}} \ast \left( {\frac{{{\,}^{13}{\mathrm{C}}}}{{{\,}^{12}{\mathrm{C}}}}} \right)_{\mathrm{B}} + {\mathrm{C}} \ast \left( {\frac{{{\,}^{13}{\mathrm{C}}}}{{{\,}^{12}{\mathrm{C}}}}} \right)_{\mathrm{C}}$$

and

3 $$1/\left( {\frac{{\mathrm{CO}_2}}{{{\,}^3{\mathrm{He}}}}} \right)_{\mathrm{Obs}} = {\mathrm{A}}/\left( {\frac{{\mathrm{CO}_2}}{{{\,}^3{\mathrm{He}}}}} \right)_{\mathrm{A}} + {\mathrm{B}}/\left( {\frac{{\mathrm{CO}_2}}{{{\,}^3{\mathrm{He}}}}} \right)_{\mathrm{B}} + {\mathrm{C}}/\left( {\frac{{\mathrm{CO}_2}}{{{\,}^3{\mathrm{He}}}}} \right)_{\mathrm{C}}$$

where A, B and C refer to three different components and A+B+C = 1.

The predicted δ^13^C_CO2_ produced by the acid dissolution of the Marble Delta Formation between temperatures of 10 and 100 °C, depicted on Fig. 2, was calculated from the measured Marble Delta Formation δ^13^C_carb_^20^ using established fractionation factors between δ^13^C_carb_ and gaseous CO_2_, calculated according to equation [4]^63^, where *T* is the temperature in Kelvin:

4 $$10^3ln\alpha = - 2.988 \ast \left( {10^6/T^2} \right) + 7.6663 \ast \left( {10^3/T} \right) - 2.4612$$

## Supplementary information

Supplementary Information

Peer Review File

## Data Availability

All data used to generate the figures are provided in Supplementary Tables 1–4, and Supplementary Figs. 1–3.
