# Author Correction: Noble gases confirm plume-related mantle degassing beneath Southern Africa

**DOI:** 10.1038/s41467-023-40780-2

**Published:** 2023-08-24

**Authors:** S. M. V. Gilfillan, D. Györe, S. Flude, G. Johnson, C. E. Bond, N. Hicks, R. Lister, D. G. Jones, Y. Kremer, R. S. Haszeldine, F. M. Stuart

**Affiliations:** 1 School of GeoSciences, University of Edinburgh, Grant Institute, James Hutton Road, Edinburgh, EH9 3FE UK; 2 Isotope Geosciences Unit, Scottish Universities Environmental Research Centre, East Kilbride, G75 0QF UK; 3 Department of Civil and Environmental Engineering, University of Strathclyde, James Weir Building, Glasgow, G1 1XJ UK; 4 School of Geosciences, University of Aberdeen, Meston Building, Kings College, Aberdeen, AB24 3UE UK; 5 Council for Geoscience, 139 Jabu Ndlovu St., Pietermaritzburg, KwaZulu-Natal 3200 South Africa; 6 British Geological Survey, Environmental Science Centre, Nicker Hill, Keyworth, Nottingham, NG12 5GG UK; 7 Present Address: Department of Earth Sciences, University of Oxford, 3 South Parks Rd, Oxford, OX1 3AN UK

Correction to: *Nature Communications* 10.1038/s41467-019-12944-6, published online 5 November 2019

The original version of the Supplementary Information associated with this Article included tracked changes. The HTML has been updated to include a corrected version of the Supplementary Information without tracked changes.

### Supplementary information

Updated Supplementary Information

