# Supplementary material for: Noble gases confirm plume-related mantle degassing beneath Southern Africa
Source: Nat Commun. 2019 Nov 5;10:5028. doi: 10.1038/s41467-019-12944-6 (PMC6831580; doi:10.1038/s41467-019-12944-6)
Supplement: Supplementary file 1 — Supplementary Information [file 41467_2019_12944_MOESM1_ESM.docx]

1. **Supplementary Information for Gilfillan et al., Noble gases confirm plume related**
2. **mantle degassing beneath Southern Africa**

3

1. **Supplementary Table 1:** *List of samples and sample locations depicted on Fig. 1.* Water
2. temperature in °C, pH, Total Dissolved Solids as measured in the field using a Hanna
3. Instruments 991301 portable EC, TDS, pH and temperature meter. A description of the seep
4. morphology is also provided.

Sample Name

Location (Latitude, Longitude)

Temperature (°C)

pH TDS

(ppt gl^-1^)

Seep description

A - Baker Farm -30.69472,

30.04166

Dry well - no water present

Partially sealed but active gas venting well bore, originally drilled as a groundwater well.

|  | B - Mjaja | -30.75388, | 23.5 | 6.95 | 6.29 | Bubbling seep in stream |
| --- | --- | --- | --- | --- | --- | --- |
|  |  | 29.97305 |  |  |  |  |
|  | C - Umtamvuna | -30.80722, | 20.9 | 5.41 | 2.31 | Seep emitting from a 100 m |
|  | Mound 2 (UM) | 29.96222 |  |  |  | wide, 30 m high travertine mound. No evidence of recent precipitation. |
|  | C - Umtamvuna | -30.80861, | 20.2 | 5.56 | 2.81 | Stream of bubbles seeping from |
|  | River Spring (URS) | 29.96250 |  |  |  | the river |
|  | C- East Cape | -30.80861, | 20.5 | 6.22 | 3.29 | Bubbling spring contained |
|  | Small Travertine (ECST) | 29.96250 |  |  |  | within a 1m diameter travertine cone |
|  | C - East Cape | -30.80819, | 20.1 | 6.24 | 3.18 | Bubbling spring contained |
|  | Large Travertine (ECLT) | 29.96224 |  |  |  | within a 2.5 m diameter travertine cone |
| 8 |  |  |  |  |  |  |
| 9 |  |  |  |  |  |  |

1. **Supplementary Table 2:** *Measured noble gas concentrations in cm^3^(STP)cm^-3^*. 1σ errors to
2. last significant figure are provided in brackets, with bracketed letters for sample localities
3. corresponding to Fig. 1.

Sample δ13CCO2 (‰) 4He (10^-6^) ^20^Ne (10^-8^) ^40^Ar (10^-5^)

(UM)

| A - Baker Farm 1 -2.0 (2) 96.2 (46) | 4.13 (18) | 7.83 (29) |
| --- | --- | --- |
| B - Mjaja -2.0 (2) 26.6 (13) | 1.19 (5) | 3.71 (14) |
| C - Umtamvuna Mound 2 -3.4 (2) 0.00480 (15) | 0.401 (2) | 0.634 (24) |
| C - East Cape Large Travertine -2.9 (2) 0.713 (35) | 1.75 (7) | 2.87 (11) |
| C - East Cape Small Travertine -3.3 (2) 0.290 (9) | 2.82 (10) |  |
| C - Umtamvuna River Spring 0.164 (5) | 2.49 (9) |  |

(ECLT)

(ECST) (UM-RS)

13

1. **Supplementary Table 3:** *Noble gas isotope ratios, CO2/^3^He and stable isotope values*. ^3^He/^4^He are reported relative to the atmospheric ratio
2. (RA: 1.399 × 10^-6^ after^37^). Rc = measured ^3^He/^4^He corrected for air contamination using equation [1]. δ^13^CCO2‰ = [(^13^C/^12^Csample-
3. ^13^C/^12^Cstandard)/(^13^C/^12^Cstandard)] x 1000; the standard used is the Vienna PeeDee Belemnite. 1σ errors to last significant figure provided in brackets
4. with the letters for sample localities corresponding to Fig. 1.

|  | Sample | 3He/^4^He  (Rm/RA) | 3He/^4^He  (Rc/RA) | 20Ne/^22^Ne | 21Ne/^22^Ne | 40Ar/^36^Ar | 38Ar/^36^Ar | 4He/^20^Ne | CO2/^3^He  (10^10^) |
| --- | --- | --- | --- | --- | --- | --- | --- | --- | --- |
|  | A - Baker Farm 1 - MAP | 3.92 (5) | 3.9 (3) | 9.98 (3) | 0.0304 (2) | 961 (4) | 0.191 (4) | 2329 (151) | 0.188 (10) |
|  | B - Mjaja 1 - MAP | 3.93 (4) | 3.9 (3) | 9.89 (3) | 0.0300 (2) | 549 (2) | 0.189 (4) | 2229 (144) | 0.678 (35) |
|  | C- Umtamvuna Mound 2 (UM) | 2.80 (2) | 3.6 (2) | 9.79 (3) | 0.0292 (2) | 298 (3) | 0.182 (7) | 1.19 (7) | 5238 (474) |
|  | C - East Cape Large Travertine (ECLT) | 3.58 (4) | 3.6 (2) | 9.77 (3) | 0.0292 (2) | 302 (2) | 0.187 (3) | 41.0 (26) | 27.7 (14) |
|  | C - East Cape Small Travertine (ECST) | 3.95 (7) | 4.1 (2) | 9.79 (3) | 0.0288 (2) |  |  | 10.3 (5) | 61.8 (22) |
|  | C- Umtamvuna River Spring (UM-RS) | 4.27 (8) | 4.5 (2) | 9.76 (3) | 0.0286 (2) |  |  | 6.59 (30) | 101 (4) |
| 18 |  |  |  |  |  |  |  |  |  |
| 19 |  |  |  |  |  |  |  |  |  |

1. **Supplementary Table 4:** *Results of the high precision analysis of Ne isotopes performed on the ARGUS mass spectrometer.* ^20^Ne/^22^Ne,
2. ^21^Ne/^22^Ne uncorrected for the contribution from ^20^NeH^+^ and ^21^Ne/^22^Ne corrected for the contribution from ^20^NeH^+^ are provided. 1σ errors to last
3. significant figure provided in brackets with the letters for sample localities corresponding to Fig. 1.

|  | Sample | 20Ne/^22^Ne | 21Ne/^22^Ne uncorrected for ^20^NeH^+^ | 21Ne/^22^Ne corrected for ^20^NeH^+^ |
| --- | --- | --- | --- | --- |
|  | A - Baker Farm 2a - ARGUS | 9.959 (7) | 0.02996 (7) | 0.02966 (8) |
|  | A - Baker Farm 2b - ARGUS | 9.961 (7) | 0.03003 (5) | 0.02976 (6) |
|  | B - Mjaja 2a - ARGUS | 9.881 (7) | 0.03021 (5) | 0.02963 (5) |
|  | B - Mjaja 2b - ARGUS | 9.892 (5) | 0.03013 (5) | 0.02956 (5) |
| 23 |  |  |  |  |


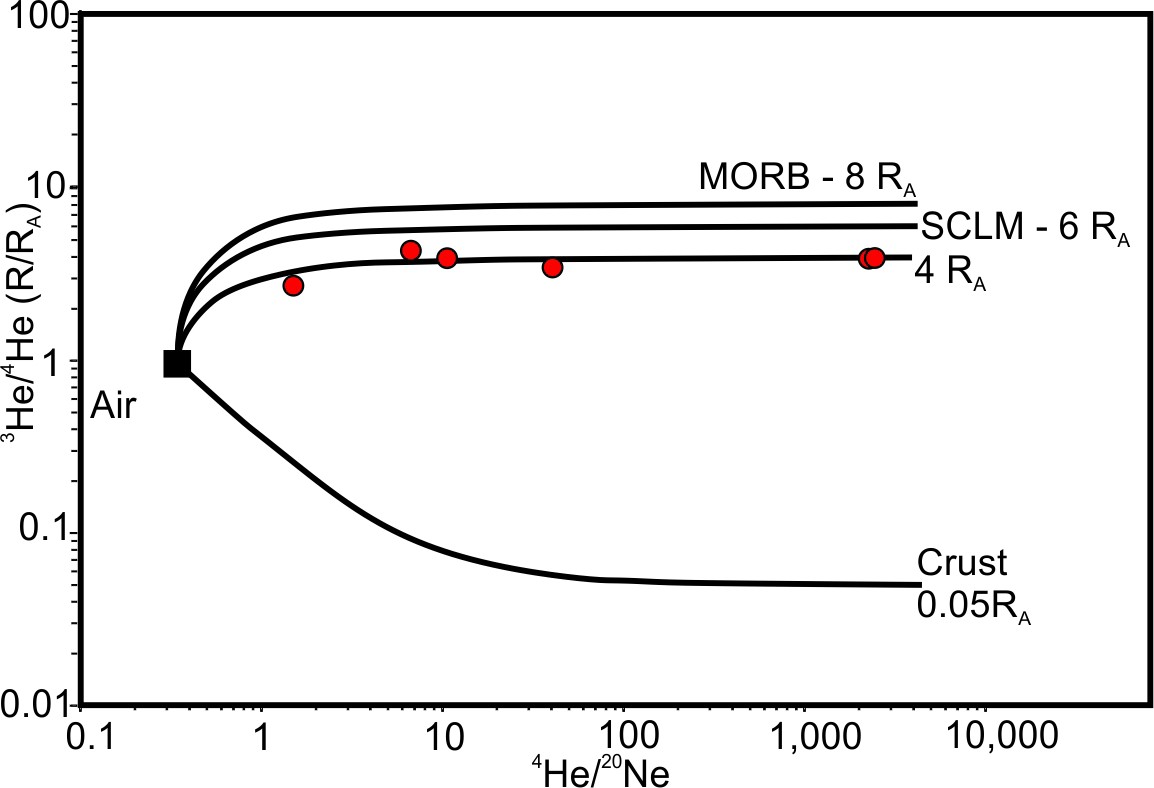


**Supplementary Figure 1:** *Plot of air corrected ^3^He/^4^He (R/RA) against ^4^He/^20^Ne for the Bongwan CO2 samples (red circles).* Also plotted is the atmospheric air value (black square) and mixing lines between the air value and: typical average crust (^3^He/^4^He = 0.05 RA and

^4^He/^20^Ne = >5000 ^41^), Mid Ocean Ridge Basalt (MORB) mantle (^3^He/^4^He = 8 RA and ^4^He/^20^Ne

= >5000 ^46^) and Sub-continental Lithospheric mantle (SCLM) (^3^He/^4^He = 6 RA and ^4^He/^20^Ne

= >5000 ^46^). This highlights that the ^3^He/^4^He and ^4^He/^20^Ne of the Bongwan CO2 samples can be explained by mixing between mantle, crustal and atmospheric air components with a consistent mantle-crust mixing ratio, and a ^3^He/^4^He end member of ~4 RA. All errors are smaller than printed symbols.

**10.2**


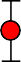

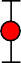

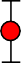

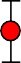


**Air**

**10.1**

**MFL**

**10.0**

**^20^Ne/^22^Ne**

**9.9**

**9.8**

**9.7**

**9.6**

**0.170 0.175 0.180 0.185 0.190 0.195 0.200**

**^38^Ar/^36^Ar**

**Supplementary Figure 2:** *Plot of ^20^Ne/^22^Ne against ^38^Ar/^36^Ar for the Bongwan CO2 samples (red circles)*. 1σ errors associated with each measurement are shown and the atmospheric air (black square) and the mass fractionation line (MFL) are also plotted. All of the Bongwan CO2 samples exhibit ^38^Ar/^36^Ar that are within error of the air value, as are the ^20^Ne/^22^Ne, other than that of Baker Farm, which exhibits ^20^Ne/^22^Ne above air and does not lie on the MFL. 1σ error envelopes are also plotted.

**10.2**


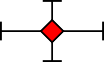

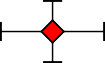

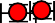

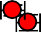

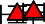

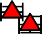


**BF**

**M**

**Air**

Iceland

Loihi-Kilauea

Réunion

**10.1**

**Solar**

Kerguelen

**10.0**

**^20^Ne/^22^Ne**

**MORB**

**9.9**

**9.8**

**Crust**

**9.7**


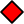

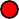

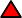


MAP uncorrected for NeH^+^ ARGUS corrected for NeH^+^ ARGUS uncorrected for NeH^+^

**MFL**

**9.6**

**0.028 0.029 0.030 0.031 0.032**

**^21^Ne/^22^Ne**

**Supplementary Figure 3:** *Plot of ^20^Ne/^22^Ne against ^21^Ne/^22^Ne for all of the measured Bongwan CO2 samples*. 1σ errors associated with each measurement are provided and the plot includes lower precision analysis uncorrected for NeH^+^ (red diamonds); higher precision analysis uncorrected for NeH^+^ (red diamonds) and the higher precision analysis corrected for NeH^+^ (red triangles). This highlights that the Mjaja sample is within the error envelope of the low precision measurement, which was not corrected for the ^20^NeH+ contribution to ^21^Ne. The uncorrected high precision Baker Farm sample plots further above the air-MORB line than the low precision measurement. This can be explained by the fact that the high precision analysis was performed on a separate sample of the CO2 from the Baker Farm well, which was collected at a later time, after the well had been sealed off from the atmosphere for a greater period of time. The approximately 1 hour period of magmatic gas accumulation in the sealed well between sample collection can account for the more mantle enriched signature of the uncorrected high precision sample.
